# When sugar takes your breath away: how diabetes accelerates COPD progression

**DOI:** 10.1038/s44321-026-00501-w

**Published:** 2026-08-17

**Authors:** Annabelle Elisabeth Kruf, Kathrin Maedler

**Affiliations:** University of Bremen, Islet Biology Laboratory, Centre for Biomolecular Interactions Bremen, Bremen, Germany

**Keywords:** Immunology, Metabolism, Respiratory System

## Abstract

K. Maedler and A. Kruf discuss the study by Chen et al in this issue of EMBO Mol Med, which shows that hyperglycemia impairs antiviral defense in the lung while promoting excessive inflammation through the accumulation of advanced glycation end products (AGEs).

**Figure:**
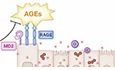

## Teaser

Diabetes is much more than high blood sugar. In this issue of EMBO metabolism, Chen et al (REF Chen et al 2026) show that hyperglycemia impairs the lung’s antiviral defense while driving excessive inflammation through the accumulation of harmful advanced glycation end products (AGEs). As viral infections are a major trigger of chronic obstructive pulmonary disease (COPD) exacerbations and can also worsen glycemic control, these findings provide a mechanistic explanation for the vicious cycle linking diabetes and COPD. By identifying AGEs as key mediators of impaired antiviral immunity, the study highlights AGE inhibition as a promising therapeutic strategy to reduce viral susceptibility and improve outcomes in patients with COPD - diabetes comorbidity.

## Three interconnected burdens: COPD, diabetes, and viral infections

COPD begins with inflammation of the small airways, and eventually leads to chronic and irreversible damage of the whole lung parenchyma and progressive dyspnea. Primarily driven by exposure to air pollution, dust and cigarette smoke, acute exacerbations are most commonly triggered by respiratory viral infections, including the influenza virus, rhinovirus, and respiratory syncytial virus. The intense airway inflammation accelerates lung function decline and increases mortality, particularly in patients with uncontrolled type 2 diabetes (T2D) (Fabbri et al, 2023). The clinical evidence is clear: diabetes is associated with worse COPD outcomes.

The COVID-19 pandemic highlighted the impact of metabolic disease on susceptibility to severe viral infections. Patients with obesity-associated metabolic dysfunction and diabetes suffered from more severe infections with higher mortality than individuals without diabetes. Multiple factors contribute to this increased risk: a persistent pro-inflammatory state, impaired immune responses, endothelial dysfunction, and chronic hyperglycemia (Maedler et al, 2002; Zhu et al, 2020). Hyperglycemia alters the body’s ability to control viral replication while simultaneously promoting excessive inflammation and tissue injury. The profound impact of diabetes on antiviral host defense, susceptibility to viral damage, and the outcome of respiratory viral infections highlights the urgent need for mechanistic insights and novel therapeutic strategies.

## AGE in RAGE: a molecular mechanism linking hyperglycemia, COPD, and viral susceptibility

The biological mechanisms underlying severe acute exacerbations of COPD (AECOPD) have remained elusive. Is the increased risk driven by systemic immune impairment, or does diabetes actively reshape the lung microenvironment to promote disease progression? Understanding this link is essential for developing targeted strategies to identify and protect vulnerable patients.

**Figure 1:**
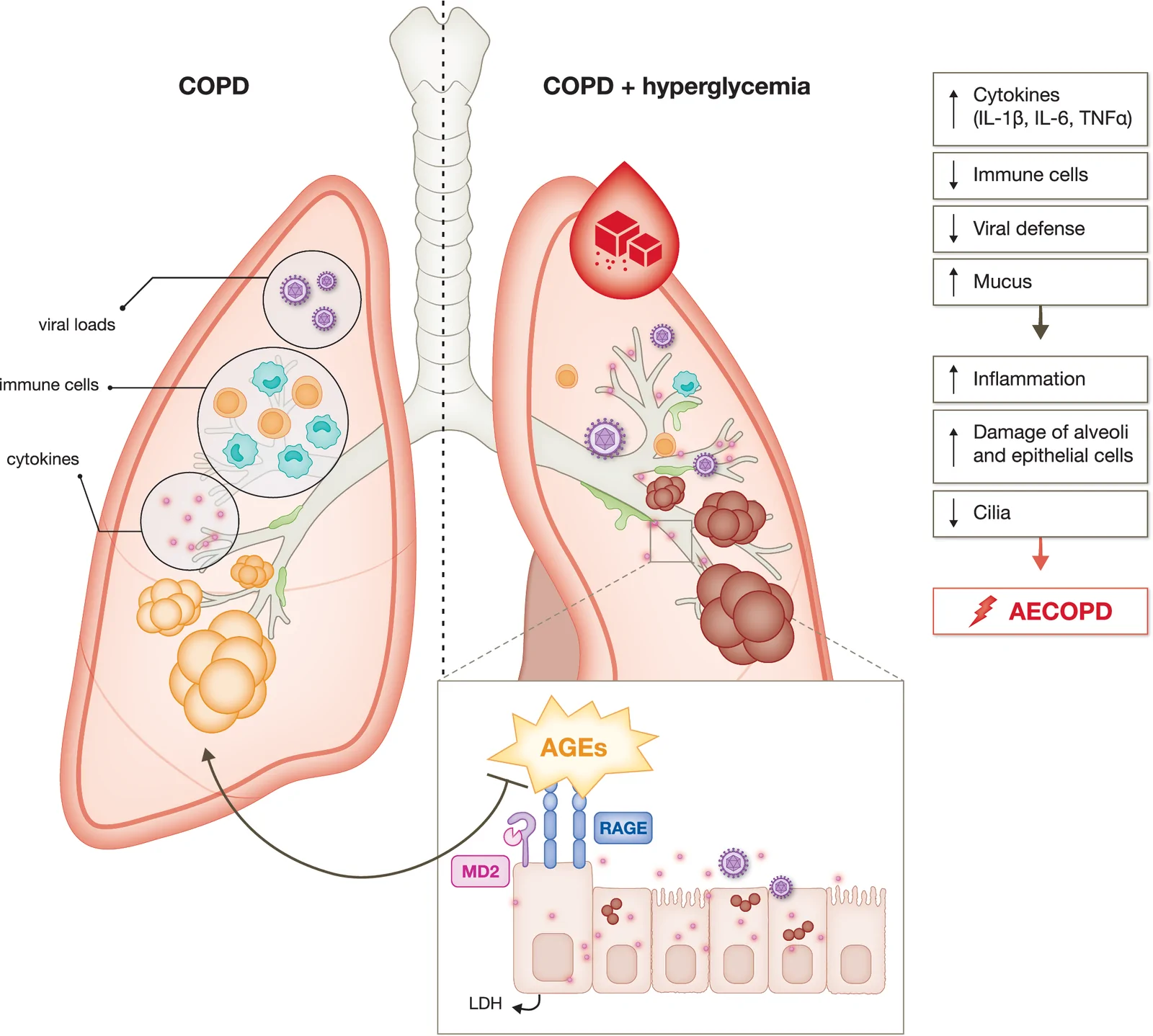
Hyperglycemia promotes viral susceptibility in COPD through the AGE–RAGE axis. While COPD is characterized by macrophage (purple) and T-cell (blue) infiltration, chronic inflammation (pink), and mucus hypersecretion (brown droplets) (left), hyperglycemia promotes the formation of advanced glycation end products (AGEs) (right). AGEs bind to RAGE and myeloid differentiation protein 2 (MD2), activating intracellular signaling pathways that reprogram the antiviral response. This shifts the inflammatory response from immune cell-driven inflammation toward exaggerated inflammation in airway epithelial cells, leading to increased viral susceptibility, higher viral loads (green), and more severe epithelial and alveolar injury, including ciliary loss and elevated lactate dehydrogenase (LDH) release. These mechanisms contribute to worse COPD outcomes in patients with hyperglycemia and diabetes and increase the risk of acute COPD exacerbations (AECOPD). Pharmacological inhibition of AGE formation, therefore, represents a promising therapeutic strategy to prevent hyperglycemia-driven AECOPD.

Chen et al 2026 performed single-cell RNA sequencing (scRNA-seq) of bronchial samples obtained during bronchoscopy and reveal a shift from inflamed immune cells to inflamed lung epithelial cells in patients with COPD and hyperglycemia. While immune cells exhibited an impaired antiviral defense program, airway epithelial cells showed marked activation of viral response and inflammatory pathways compared with patients with COPD alone (Fig. 1). This shift is likely to compromise antiviral defense. Notably, enrichment of advanced gycation end products (AGEs), their receptor (RAGE), and Toll-like receptor (TLR) signaling pathways in samples from patients with COPD-diabetes comorbidity drew attention to AGE-RAGE axis as a potential driver of AECOPD.

Chronic hyperglycemia promotes the formation of advanced glycation end products (AGEs) through the chemical modification of proteins, lipids, and nucleic acids, thereby altering their function. Unlike transient elevations in blood glucose, AGEs accumulate in tissues, and can persist long after glycemic control has improved. This phenomenon, often termed “metabolic memory”, implies that the damage sustained during periods of hyperglycemia can persist independently of current glucose levels. Through interaction with receptors such as RAGE and myeloid differentiation protein 2 (MD2), AGEs promote inflammation, oxidative stress, and tissue injury, and are well-established drivers of the development and progression of diabetes and its complications, particularly in the kidney, heart and vasculature (Chaudhuri et al, 2018).

In contrast, their role in the respiratory system has remained largely unexplored. Whereas RAGEs are typically low in the airway epithelium, Chen et al 2026 found them upregulated in the COPD airway lining, where they contribute to tissue injury. In addition, direct binding of AGEs to MD2 promotes the formation of an AGEs-MD2-TLR4 signaling complex and initiation of pro-inflammatory pathways in the lung. Similar mechanisms have been implicated in diabetes-induced inflammatory cardiomyopathy (Wang et al, 2020), as well as in TLR4-MD2-mediated inflammation in pancreatic β-cells and adipocytes under diabetic conditions.

Downstream, the AGE-RAGE-MD2 axis activates key inflammatory signaling pathways, including p38, ERK, and NFκB. Beyond promoting inflammation, this pathway compromises the lung’s antiviral defenses. Increased vulnerability to viral infection in a diabetic milieu has similarly been reported in the pancreas (Geravandi et al, 2025; Zhu et al, 2020). Consistent with this, Chen et al 2026 show that airway epithelial cells derived from patients with COPD, as well as from mouse models of COPD, exhibit marked increased susceptibility to influenza virus under hyperglycemic conditions, resulting in higher viral loads and epithelial barrier breakdown. This confirms that the viral risk is not inherent to COPD alone, but is specifically exacerbated by the metabolic stress in diabetes.

## AGE inhibitors for the therapy of COPD and diabetes

Perhaps most importantly, pharmacological inhibition of AGEs significantly reduced viral load in the lung both in vitro and in vivo. Chen et al 2026 findings open two potential avenues for clinical translation. First, elevated AGEs levels in sputum or bronchoalveolar fluid could serve as biomarkers to identify patients at increased risk of AECOPD. Second, pharmacological targeting of AGEs emerges as a promising therapeutic strategy. Small-molecule AGE inhibitors have been developed and extensively reviewed (Zeng et al, 2019). Inhaled delivery of AGEs inhibitors using biodegradable nanoparticles could improve lung retention and minimize systemic adverse effects. Already several decades ago, aminoguanidine was proposed to prevent autoimmune diabetes owing not only to its efficacy as an AGE inhibitor, but also to its relatively selective inhibition of inducible nitric oxide synthase (iNOS) (Corbett and McDaniel, 1996). Blocking AGEs may therefore not only improve metabolic health but also strengthen the lungs’ antiviral defenses, offering hope for better outcomes in this vulnerable patient population.

## Early diabetes diagnosis and glycemic control: breaking the viral cycle

Viral infections cause both acute and long-term disruption of glycometabolic control throughout the body (Montefusco et al, 2021). The resulting inflammatory response directly affects glucose metabolism through a “cytokine storm”, characterized by the release of pro-inflammatory cytokines that promote insulin resistance while damaging vulnerable pancreatic β-cells. This, in turn, reduces insulin production and further aggravates hyperglycemia. Importantly, this relationship is bidirectional: (1) hyperglycemia predisposes patients to severe viral infections, and (2) viral infection itself frequently induces stress hyperglycemia, even in individuals without diabetes.

This vicious cycle highlights the importance of early diagnosis and tight glycemic control, which require a comprehensive therapeutic approach aimed not only at lowering blood glucose but also at limiting inflammation, restoring immune and muscle function, and protecting pancreatic β cells from further damage. Chronic hyperglycemia, even at subclinical levels, will further impair β-cell function and survival by sustaining a pro-inflammatory response (Maedler et al, 2002).

The current study, which included only samples from patients with uncontrolled diabetes, clearly highlights the contribution of hyperglycemia to severe COPD. These findings suggest that early detection and effective glycemic control for patients with diabetes may reduce the risk of further inflammatory dysregulation and the development of COPD-related comorbidity.
